# The Role of Protein Kinase B Signaling Pathway in Anti-Cancer Effect of Rolipram on Glioblastoma Multiforme: An In Vitro Study

**DOI:** 10.18869/nirp.bcn.8.4.325

**Published:** 2017

**Authors:** Sara Ramezani, Mahmoudreza Hadjighassem, Nasim Vousooghi, Mansour Parvaresh, Farshid Arbabi, Naser Amini, Mohammad Taghi Joghataei

**Affiliations:** 1. Neuroscience Research Center, Department of Neurology, School of Medicine, Guilan University of Medical Sciences, Rasht, Iran.; 2. Department of Neuroscience, School of Advanced Technologies in Medicine, Tehran University of Medical Sciences, Tehran, Iran.; 3. Brain and Spinal Cord Injury Research Center, Tehran University of Medical Sciences, Tehran, Iran.; 4. Iranian National Center for Addiction Studies, Iranian Institute for Reduction of High-Risk Behaviors, Tehran University of Medical Sciences, Tehran, Iran.; 5. Department of Neurosurgery, School of Medicine, Iran University of Medical Sciences, Tehran, Iran.; 6. Department of Oncology, Faculty of Medical Sciences, Zahedan University of Medical Sciences, Zahedan, Iran.; 7. Cellular and Molecular Research Center, Iran University of Medical Sciences, Tehran, Iran.; 8. Department of Neuroscience, School of Advanced Technologies in Medicine, Iran University of Medical Sciences, Tehran, Iran.

**Keywords:** Glioblastoma multiforme, Rolipram, SC79, U87 MG cell line, Tumor-initiating cells, Akt signal

## Abstract

**Introduction:**

The mechanism of putative cytotoxicity of 4-[3-(cyclopentyloxy)-4-methoxyphenyl]-2-pyrrolidone (rolipram), a specific phosphodiesterase-4 (PDE4) inhibitor, on glioblastoma multiforme (GBM) is almost unknown. This study aimed to investigate the role of protein kinase B (Akt) pathway in the cytotoxic effect of rolipram on human GBM U87 MG cell line and Tumor-Initiating Cells (TICs) isolated from patient’s GBM specimen.

**Methods:**

TICs were characterized by using flow cytometry and quantitative real-time PCR. The cells were treated with rolipram at inhibitory concentration of 50% (IC50) in the presence or absence of SC79 (4μg/mL), a specific AKT activator, for 48 hours. The cell viability and apoptosis were measured by MTT assay and TUNEL staining, respectively. The relative expression of Phospho-Akt (Ser473), matrix metalloproteinase 2 (MMP2), and vascular endothelial growth factor A (VEGFA) were detected using Western blotting.

**Results:**

The findings showed that rolipram could suppress cell viability in both U87MG and TICs, dose-dependently. Interestingly, the rolipram-induced cytotoxicity was significantly reduced in the presence of SC79. Nevertheless, in rolipram-treated cells, the pretreatment with SC79 significantly led to increase in U87 MG cells and TICs apoptosis and decrease in viability of U87 MG cells but not TICs relative to corresponding control. In U87 MG and TICs, rolipram-induced reduction of Phospho-Akt (Ser473) and MMP2 levels were significantly suppressed by SC79.

**Conclusion:**

There is a cell type-specific mechanism of anti-proliferative action of rolipram on GBM cells. The reduction of intracellular level of MMP2 but not VEGFA by rolipram is conducted through the inhibition of Akt signal. Rolipram-induced apoptosis is mediated via Akt dependent/independent mechanisms.

## 1. Introduction

Glioblastoma Multiforme (GBM) is the most common malignant primary brain tumor in adults which is classified as grade IV astrocytoma (Wen & Kesari, 2008). Despite standard therapeutic interventions, the patients succumb to death 12–15 months after diagnosis (Furnari et al., 2007; Grossman et al., 2010; Stupp et al., 2009; Stupp et al., 2005). Recently, it has been revealed that the expression level of a brain-specific isoform of phosphodiesterase 4, PDE4A1, in GBM tissue is substantially increased. It is suggested that PDE4 plays a positive regulatory role in intra-cranial glioma growth. Low level of cyclic adenosine mono-phosphate (cAMP) has been also detected in the majority of brain tumors (Furman & Shulman, 1977; Goldhoff et al., 2008; Houslay & Adams, 2003).

Based on these findings, using PDE inhibitors to induce tumor regression has been initiated (Sengupta, Sun, Warrington, & Rubin, 2011). In the last decade, some studies have been focused on the effect of rolipram on tumor suppression in several cancer models (Chen et al., 2002; El-Mowafy & Alkhalaf, 2003; Goldhoff et al., 2008; McEwan et al., 2007; Moon, Lee, Lee, Kim, & Lee, 2012; Tsunoda et al., 2014; Warrington et al., 2010). Although, these studies have indicated the anti-tumor property of rolipram, molecular mechanisms of putative cytotoxicity of rolipram have not been well discovered, yet. Some documents suggest that the anti-cancer effect of rolipram is mediated via cAMP-dependent Protein Kinase A (PKA) (Caretta & Mucignat-Caretta, 2011; Cheng, Phelps, & Taylor, 2001). Two isozyme forms of PKA exist which are functionally different in regulatory subunit; RI and RII are growth stimulator and inhibitor, respectively. The isoform II but not I complexes demand to fill both two cAMP binding domains for high affinity interaction with catalytic subunits (Anand et al., 2007).

GBM tend to have low cAMP level and a disturbance in the homeostatic balance between PKA isoforms, leading to tumorigenesis (Chen, Hinton, Zidovetzki, & Hofman, 1998). On the other hand, several papers have demonstrated that stimulation of cAMP by its analogues dramatically reduces AKT and mitogen-activated protein kinase (MAPK) activities and leads to apoptosis in cancer cell lines (Ahn, Jung, & Hong, 2005; Tsunoda et al., 2012; Wang, Liu, & Adamo, 2001). AKT, a serine threonine kinase, is key regulator of intracellular processes promoting the cell growth, migration, and survival (Engelman, 2009). Several studies have signified that tumor angiogenesis and invasion are crucial processes to progress GBM. These processes are triggered via abundant secretion of vascular endothelial growth factor (VEGF) and matrix metalloproteinase (MMPs) by tumor cells (Bao et al., 2006; de Groot et al., 2010; Folkins et al., 2009).

Likewise, it has been discovered that activation of AKT pathway stimulates VEGF and MMPs production by cancer cells and pharmacological inhibition of AKT can suppress the expression of VEGF and MMP2 (Karar & Maity, 2011; Kwiatkowska, Kijewska, Lipko, Hibner, & Kaminska, 2011; Tsou et al., 2013). Although, previous research clarified that PDE4 inhibitor downregulates expression of VEGF and MMPs (Martin-Chouly et al., 2004; Pullamsetti et al., 2013), nevertheless it is unclear whether these downregulations due to PDE4 inhibitor are mediated through AKT pathway. Therefore, more studies are required to realize the underlying mechanism of action of rolipram to induce the cytotoxicity in vitro on human GBM cell lines and tumor-initiating cells (TICs).

TICs display an extremely invasive phenotype and contribute to tumor growth and angiogenesis. Moreover, TICs are resistant to radiation and chemotherapy (Cheng, Bao, & Rich, 2010). Therefore, therapeutic targeting of TICs to achieve cure and reduce tumor relapse has clinical significance. Thus, we set out to determine the inhibitory concentration 50% (IC50) of rolipram in U87 MG and TICs cultures and then investigate whether AKT pathway contributes in rolipram-induced cytotoxicity. We also evaluated the role of AKT pathway in modulation of intracellular VEGFA and MMP2 levels by rolipram.

## 2. Methods

### 2.1. Reagents

Rolipram, SC79 AKT activator, mehtylthiotetrazole (MTT), DNase I, dimethylsulfoxide (DMSO), and Phosphate-Buffered Saline (PBS) were purchased from Sigma-Aldrich. Dulbecco’s Modified Eagle Medium/nutrient mixture F-12 (DMEM/F-12) and trypsin, 0.25% (1x) with EDTA were purchased from Gibco; Life Technologies. Trypsin Inhibitor Soybean, antibiotic/antimycotic, penicillin/streptomycin, Epidermal Growth Factor (EGF), basic fibroblast growth factor (b-FGF), B27, heparin and fetal bovine serum (FBS) were bought from Invitrogen.

### 2.2. U87 MG cell line culture

Human glioblastoma U87MG cell line (C531) was purchased from Pasteur Institute of Iran and maintained in DMEM/F-12 culture medium supplemented with 10% FBS and 1% penicillin/streptomycin. The cells were expanded at 37°C, 5% CO_2_ in appropriate culture flasks. The cells were replated every 4 days. The cells at passage 5 were used for the subsequent experiments.

### 2.3. GBM tissue sampling

The tissues of tumor mass core and periphery were provided from a patient diagnosed with primary glioblastoma who did not undergo prior chemotherapy or radiotherapy regimens. Study protocol was approved by the Ethics Committee of Tehran University of Medical Sciences. The tumor periphery and core tissues were surgically resected and approximately 4 mm of them separately inserted inside liquid nitrogen or the micro-tubes containing RNA later solution and stored at −80°C for further molecular assay. The rest tissues of core were placed in proper falcon tubes contained DMEM/F-12 supplemented with 10% antibiotic/antimycotic and then transported to the lab.

### 2.4. Tissue digestion and primary culture of TICs

To culture tumor initiating cells, the tissue of tumor core was washed with PBS and cut into small pieces using a No. 10 scalpel blade and subjected to enzymatic dissociation via incubation with DNase I (1.5mg/mL) and trypsinization for 15 minutes at 37°C. Then the equal volume of trypsin inhibitor soybean was added to the solution. After pipetting up and down, it was centrifuged at 1200 rpm for 5 min. The supernatant was removed and the pellet was filtered through a 70-micron cell strainer. Single cells were plated at density of 5×10^4^ cells/mL in non-adherent appropriate cell culture flasks consisting of serum-free medium supplemented with B27(5X) at a 5:1 ratio, 20 ng/mL EGF, 20 ng/mL b-FGF, 2 μg/mL heparin and 1% antibiotic/antimycotic.

Then, the cultures were incubated at 37°C in a humidified chamber with 5% CO_2_. Growth factors were replenished every 2 days. Primary glioma spheres were formed 3 days following initial culture. The spheres were monitored using an inverted microscope. The spheres were replated when their average size became 200 μ in diameter within 2 weeks. The Spheres at passage 5 were used for the subsequent experiments.

### 2.5. Characterization of TICs

TICs were characterized using flow cytometry and quantitative real-time PCR (qPCR). CD133 has been identified as a powerful marker of TICs (Hadjipanayis & Van Meir, 2009). CD133 has been defined as a prognostic indicator for tumor recurrence (Liu, et al., 2006; Ogden, et al., 2008; Singh, et al., 2004). CD15 is known as a strong marker to characterize TICs. High expression of CD15 has been also found in brain tumors particularly GBM.

The expression of this marker is linked to tumor propagation (Son, Woolard, Nam, Lee, & Fine, 2009). Thus, we decided to examine the expression of CD133 and CD15 on core-derived neurospheres of glioma in primary culture. CD133 and CD15 as positive markers and CD34 and CD45 as negative markers were considered. Dissociated and non-adherent neurospheres derived from GBM in primary culture were harvested and re-suspended in PBS and then labeled with anti-CD133 FITC-conjugated antibody (Abcam), anti-CD15 phycoerythrin (PE)-conjugated antibody (Abcam), anti-CD45 FITC-conjugated antibody (Abcam) and anti-CD34 PE-conjugated antibody (Abcam) for 60 min at 4°C in dark.

The cells were stained with FITC and PE conjugated mouse IgG1 antibodies (Santa Cruz) were assumed as isotype controls. The cells were monitored using BD FACSCalibur flow cytometer device (BD Biosciences, USA). Plentiful production of VEGFA by TICs has also been manifested (Singh et al., 2003). Thus, we detected the endogenous level of VEGFA mRNA in the core and periphery of tumor mass. Initially, total RNA of the core and periphery tissues was extracted using RNeasy Tissue Mini Kit (Qiagen Group). To synthesize the first strand complementary DNAs (cDNAs), reverse transcription of mRNA (1 μg) was done using QuantiTect Reverse Transcription Kit (Qiagen).

The oligonucleotide reverse and forward primers applied for real-time PCR amplification of the target genes, consist of beta-actin (housekeeping gene) and VEGFA, were provided by Qiagen company primer bank. Power SYBR_ Green PCR Master Mix (Life Technologies, Grand Island, NY, USA) in a final volume of 25 μL was utilized to carry out the real-time PCR reactions on StepOnePlus real-time PCR System (Applied Biosystems, Foster City, CA, USA). The volume of cDNA in all reactions was 2 μL. The annealing temperature for all genes was optimized at 60°C. Standard curve method was utilized to quantify the target genes in all samples. The Ct values of samples were determined based on the standard curve. In order to normalize the data of all samples, beta-actin was exploited as the reference gene. The amplicons length confirmation was obtained by visualizing the PCR products on 2.5% agarose gel. The specificity of reactions was confirmed by melting curve analysis depicting one single peak for each gene. Real-time PCR reactions for all samples were performed in duplicate. The analysis was done on the mean data.

### 2.6. Treatments

The cells at passage 5 were harvested and the numbers of 2.5×10^4^ cells/well were seeded in 24-well plates containing serum-free DMEM-F12. Twenty four hours later, the cells were treated with increasing concentrations of RLP (30, 100, 300, 1000 μM) for 48 h. All treatments were done in quadruplicate. After finding of IC50 of rolipram, the experiments were continued based on IC50 value of rolipram. DMSO with the same volumes was used instead of RLP, as control. SC79 at concentration of 4 μg/mL, which significantly activates AKT phosphorylation within 5 min, was added to cultures at 30 min before starting treatment with rolipram.

### 2.7. Cell viability measurement using MTT assay

Following serum starvation for 24 h, U87 cell line in monolayer culture and TICs in primary culture were subjected to incubate with drugs as described above. At the end of treatments, the medium was removed and the cells were incubated with MTT solution (5 mg/mL) for 4 h. Then, the medium was carefully discarded and formazan crystals were dissolved in 500 μL DMSO. The suspended spheres in primary culture were pelleted down by centrifugation. Optical Density (OD) was read at 570 nm by microplate reader (BioTek, USA). To calculate the cell viability rate, average value of quadruplicate absorbance readings for each treatment group were measured and normalized based on the control.

### 2.8. Cell apoptosis measurement using TUNEL staining

Terminal deoxynucleotidyl transferase dUTP nick end labelling (TUNEL) staining was carried out according to the manufacturer’s instructions (In Situ Cell Death Detection Kit, Fluorescein – Roche applied science). Briefly, the numbers of 1×10^4^ cells/well were seeded in 96-well plates. Twenty four hours later, the cells were fixed by fresh 4% paraformaldehyde at room temperature for 1 h. The cells were rinsed with PBS twice and incubated with 0.1% Triton X-100 solution in 0.1% sodium citrate at 8°C for 2 min. After washing, the cells were incubated with 50 μL TUNEL reaction mixture at 37°C in a humidified chamber for 60 min. The cells were visualized using Olympous IX71 inverted microscope, after nuclei staining with propidium iodide (PI).

For suspended cell spheres, V-bottomed 96-well plates were used. Cell suspension was centrifuged at 1200 rpm for 5 min after each washing. The plates were placed on a shaker during fixation to avoid cell clumping. TUNEL quantification was performed by calculating the percentage of TUNEL-positive cells of each field relative to total cells (PI staining) of the same field using Image J software.

### 2.9. Cyclic AMP measurement

Correlate-EIATM Cyclic AMP Enzyme Immunoassay Kit (Assay designs, Inc. USA) was used to measure the levels of cAMP. The procedures were done according to the manufacturer’s instructions. Briefly, the cells were lysed by adding 0.1 M HCl to the samples for 10 min. After centrifugation at >600×g for 10 min, the lysates were taken off. Afterward, the supernatants were resus-pended in cAMP assay buffer (Assay Designs) to run the assay procedure. All experiments were done in duplicate. At the end of assay procedure, all reactions were stopped by adding 50 μl of stop solution to each well. The plate immediately was located on the microplate reader and optical density at 405 nm was read, and cAMP concentrations were calculated based on a standard curve.

### 2.10. Protein expression measurement using western blots

The cells were collected and washed twice with PBS then centrifuged at 4000 rpm for 5 min. Cells were lysed in RIPA (Cell Signaling Technology, Netherlands) supplemented with protease inhibitor and phosphatase inhibitor cocktails (Roche, Switzerland) on ice for 30 min. Then, the mixture was centrifuged at 13000 g for 20 min at 4°. To determine Protein concentration, Bradford method was used. To do western blotting, equal values of proteins were loaded on 10% SDS polyacrylamide gel and separated in a size manner by electrophoresis. Then, proteins were transferred onto nitrocellulose membrane. Equal loading of protein was verified by Ponceau staining. The membrane was blocked with 5% fat free dry milk in Tris-buffered saline (TBS; pH=7.4) on a shaker for 1 h at room temperature. Membrane incubated with primary antibodies for PDE4A, β-actin, VEGF, MMP2, BCL-2, cleaved-caspase3, AKT1 and phosphorylated AKT (Ser473) overnight at 4°C.

Membrane was washed with TBS plus 0.5% Tween 20 (TBST) 3 times and then incubated with anti-rabbit secondary antibody conjugated with horseradish peroxidase (HRP) for 1 h at room temperature. All antibodies were obtained from Abcam except the antibody for VEGF (Santa Cruz Biotechnology) and diluted according to the manufacturer’s instructions. After washing, immunoreactive bands were visualized using an ECL detection system (Amersham Biosciences, USA). X-ray films were scanned, and then the relative protein levels were quantified by densitometry using Total lab software (BioStep). Beta-actin was tested as the internal control.

### 2.11. Statistical analysis

Concentration of rolipram in which 50% cell growth was inhibited (IC50) was determined using Prism Graph pad 6 software. All data were analyzed using IBM SPSS software (version 22.0) and the results were presented as mean±standard error of mean (SEM). The graphs were plotted using prism graph pad 6. In order to compare the difference in relative value of PDE4 protein expression between periphery and core cells of the tumor, independent samples t test was done. Relative Expression Software Tool (REST)-XL version 2 (Pfaffl, Horgan, & Dempfle, 2002) was applied to analyze the mean normalized data of VEGFA mRNA expression. Herein, pairwise fixed reallocation randomization test was utilized to compare the significant differences in relative expression levels of VEGFA between periphery and core tissues. The differences in mean normalized % viability value, relative % apoptosis value, intracellular cAMP value and relative expression values of target proteins among experimental groups were analyzed using 1-way analysis of variance (ANOVA) and Tukey as post hoc test (to evaluate specific group comparisons) in U87 MG and TICs cultures, separately. Differences were considered statistically significant at P<0.05^*^, P<0.01^**^, P<0.001^***^.

## 3. Results

### 3.1. Characterization of TICs isolated from GBM tissues

**Figure 1:**
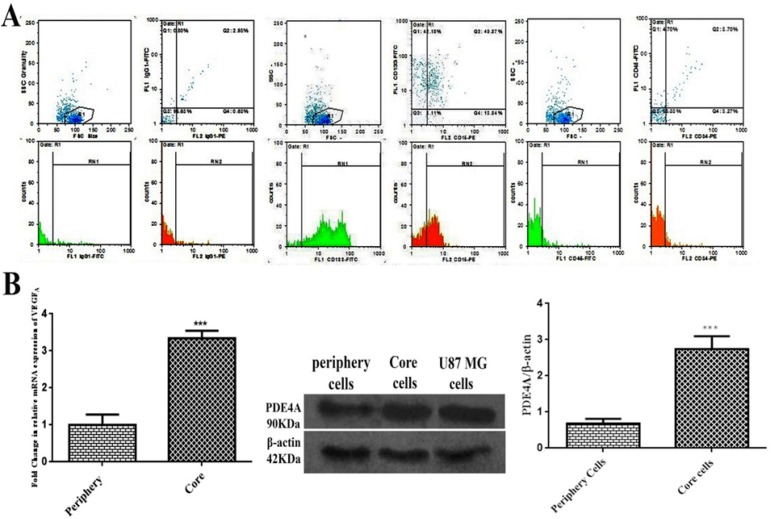
Characterization of TICs isolated from GBM tissue. (A) Flow cytometry results of GBM-derived neurospheres in primary culture. TICs were positive for CD133 and CD15 but did not considerably express CD34 and CD45. (B) The relative expression of VEGFA mRNA measured by qPCR and immunoreactive bands and relative expression of PDE4A detected by western blot in the periphery-derived cells and core-derived TICs. U87 MG cells express PDE4A protein. Beta-actin was considered as housekeeping. Bars represent fold differences of mean normalized expression values±SEM (n=3). ***P<0.001 compared to periphery zone.

The results of flow cytometry analysis elucidated that about 5%, 83%, and 54% of the total population expressed IgG1, CD133 and CD15, respectively. Collectively, almost 97% of the total population represented positive markers defining TICs subpopulation. Total cell population slightly represented CD45 and CD34 by approximately 8% and 7%, successively. These findings exhibited lack of subpopulations of endothelial and hematopoietic precursors into primary culture of core-derived neurospheres of glioma (Figure 1 A). As shown in Figure 1B, the result of qPCR illustrated that relative expression of VEGFA mRNA in TICs-enriched core zone was 3.5 fold more than peripheral zone. Relative expression of PDE4A protein in core-derived TICs was 2.5 fold more than periphery-derived cells of glioma. It was also disclosed that U87 MG cells expressed PDE4 protein.

### 3.2. The effect of rolipram at various concentrations on the viability in GBM cells

**Figure 2:**
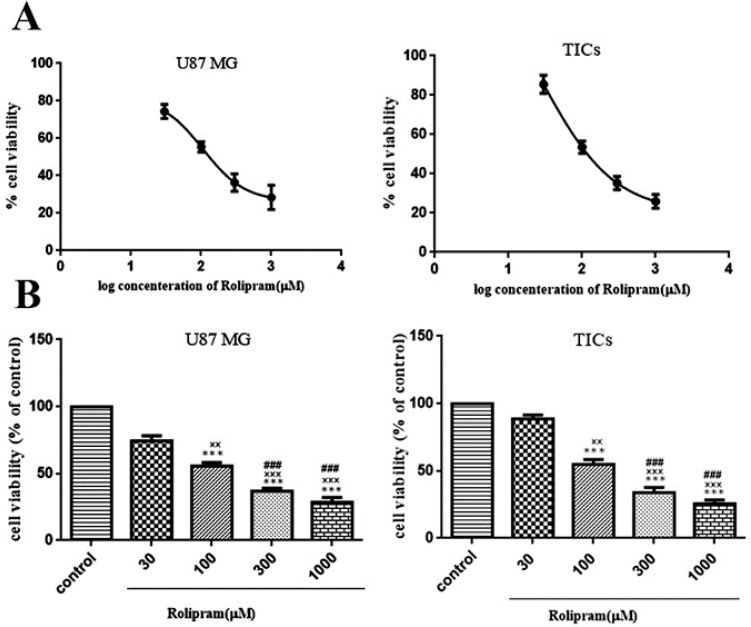
The MTT assay results and effects of indicated concentrations of rolipram on the cell viability are presented. (A) By using prism graph pad 6, the curves of log IC50 of rolipram in the U87 MG cells and TICs are illustrated. (B) The results of 1-way ANOVA analysis to compare fold differences of mean normalized % viability values among various concentrations of rolipram together and relative to control (DMSO) in U87 MG cells and TICs. Bars represent fold differences of mean normalized % viability values±SEM (n=4). ***P<0.001 compared to corresponding control, ^×××^P<0.001, ^××^P<0.01 compared to concentration 30 μM of rolipram, ^###^P<0.001 compared to concentration 100 μM of rolipram.

Rolipram diminished the number of living cells in both U87MG cells and TICs in a dose-dependent manner. According to non-linear regression analysis results, TICs and U87 MG cells were responsive to rolipram with IC50 value of ∼103.9 μM (95%CI=53.18–203.0) and ~106.8 μM (95%CI=53.98–211.5), respectively (Figure 2 A). The results of 1-way ANOVA and Tukey post hoc test demonstrated that at all tested concentrations of rolipram but 30 μM concentration, the mean % viability value significantly decreased relative to control (for U87 MG cells: at 30 μM P=0.052, at others P<0.001; for TICs: at 30μM P=0.068, at others P<0.001). It was also revealed that there was significant difference regard to mean % viability value among various concentrations of rolipram in both U87 MG and TICs cultures. The results of Tukey post hoc analysis showed that mean % viability value at 30 μM of rolipram was significantly higher than one at 100 μM (for both U87 MG and TICs P<0.01), 300 μM, and 1000 μM of rolipram (for both U87 MG and TICs P<0.001). The mean % viability value at 100 μM of rolipram was significantly different to one at 300 μM (for U87 MG P<0.003 and TICs P<0.002) and 1000 μM of rolipram (for both U87 MG and TICs P<0.001). The difference in mean % viability value between concentrations 300 μM and 1000 μM of rolipram was not statistically significant (for U87 MG P=0.211 and TICs P=0.119) (Figure 2B).

### 3.3. The effect of SC79 at various concentrations on AKT phosphorylation level in GBM cells

**Figure 3:**
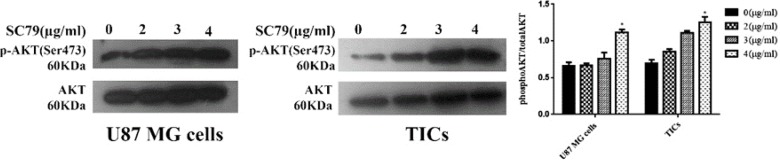
The relative expression of phosphorylated AKT (Ser473) in U87 MG cells and TICs treated with indicated consentrations of SC79 for 5 min. Bars represent fold differences of mean normalized expression values±SEM (n=4). *P<0.05 compared to untreated control (concentration 0).

Mean relative quantities of phospho-AKT (Ser473) for several concentrations of SC79 (0, 2, 3, 4 μg/mL) were depicted in Figure 3. It was revealed that incubation of the cells with 4 μg/mL of SC79 for 5 min significantly increased AKT phosphorylation (Ser473) in both U87 MG cells and TICs relative to untreated control (concentration 0) (P<0.05).

### 3.4. The effect of SC79 on the cell apoptosis and viability rates in rolipram-treated cultures

**Figure 4:**
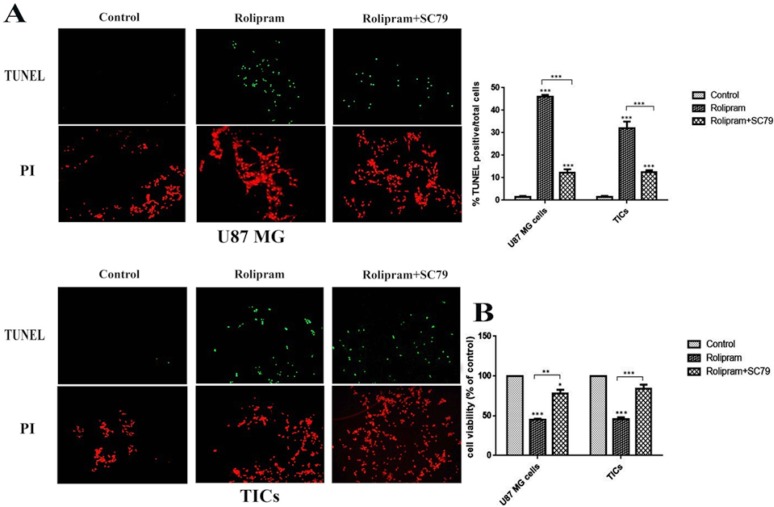
Apoptosis (A) and viability (B) rates in U87 MG cells and TICs treated with rolipram at IC50 in the absence or presence of SC79 (4μg/mL) and DMSO as control for 48 h. (A) TUNEL (green) and PI (red) positive cells were visualized at wave length of 540 and 580 nm, respectively. Bars represent mean % apoptosis values±SEM (n=4). ***P<0.001 compared to control. (B) Bares represent fold differences of mean normalized % viability values±SEM (n=4). One-way ANOVA and Tukey post hoc test were used.*P<0.05, **P<0.01, ***P<0.001 compared to control.

According to Figure 4A, rolipram at IC50 dramatically increased mean apoptosis percentage in U87 MG cells (45.92±0.5) and TICs (32.04±1.07) in comparison with corresponding control (U87 MG cells: 1.48±0.24, P<0.001; TICs: 1.44±0.24, P<0.001). IC50 of rolipram at the presence of SC79 (4 μg/mL) significantly enhanced mean apoptosis percentage in U87 MG cells (12.19±0.91) and TICs (12.61±0.41) in comparison with corresponding control (P<0.001).

Interestingly, data showed that apoptosis induction caused by rolipram at IC50 was significantly more in the absence of SC79 than that in its presence (for U87 MG and TICs P<0.001). Based on our results, rolipram at IC50 significantly reduced mean cell viability percentage relative to control (U87MG cells: 45.27±0.86, P<0.001; TICs: 45.91±4.4, P<0.001). SC79 (4 μg/mL) resulted in a significant decrease in rolipram-induced reduction of cell viability in U87 MG cells (P<0.012) and TICs (P<0.004). However, the pretreatment with SC79 (4 μg/mL) significantly lessened mean cell viability percentage in rolipram-treated U87 MG cells (77.94±9.78, P<0.041) but not in TICs (84.13±9.99, P<0.099) relative to corresponding control (Figure 4B).

### 3.5. The effect of rolipram in the presence or absence of SC79 on intracellular levels of cAMP and relative expression of cleved-caspase3, BCL-2, p-AKT (Ser473), MMP2 and VEGFA

**Figure 5:**
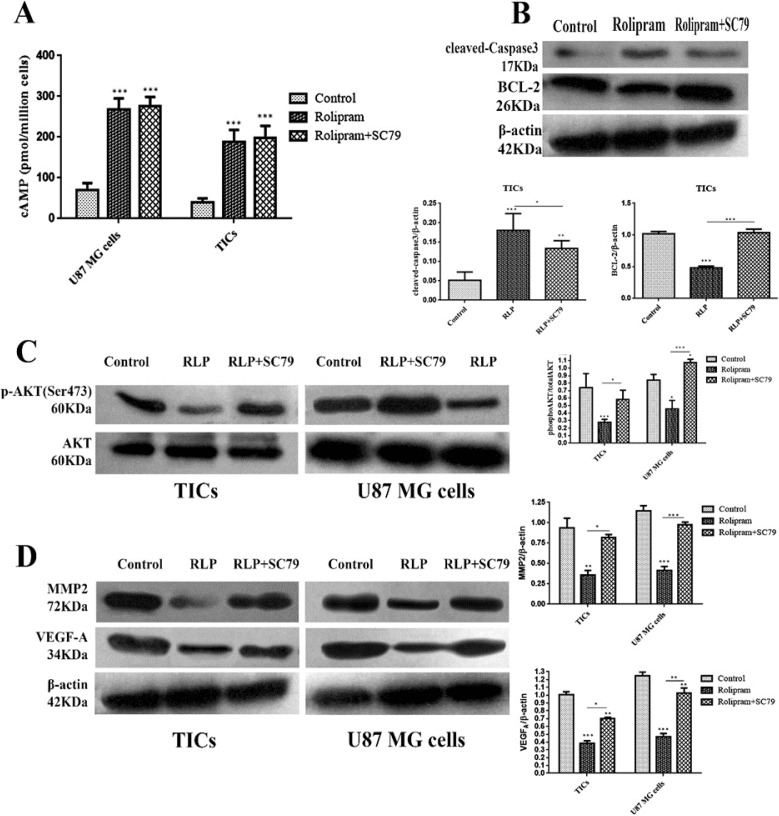
The intracellular level of cAMP determined by ELISA (A) and relative expression of cleaved-caspase3 and BCL-2 in TICs (B), phosphorylated AKT (Ser473) (C), MMP2 and VEGFA (D) detected by western blots in U87 MG cells and TICs treated with rolipram at IC50 in absence or presence of SC79 (4μg/mL) and DMSO as control for 48 h. Bars represent mean normalized values±SEM (n=4). One-way ANOVA and Tukey post hoc test were used *P<0.05, **P<0.01, ***P<0.001 compared to control.

The results illustrated that rolipram in the absence or presence of Sc79 markedly increased cAMP level in U87 MG cells and TICs relative to control (P<0.001) (Figure 5A). Western blot analysis demonstrated that rolipram in both absence (P<0.001) or presence (P<0.01) of SC79 increased relative value of cleaved-caspase3 expression in TICs compared to vehicle control. The pairwise comparison of the effects of rolipram in the absence of SC79 versus its presence indicated that the prior induced a more prominent increase in relative expression of cleaved-caspase3 (P<0.041), in the agreement with the more evident apoptosis-stimulatory effect of rolipram in the absence of SC79. We found that rolipram downregulated BCL-2 level of TICs compared to vehicle control (P<0.001). This alteration was counteracted by SC79 (Figure 5B).

It was revealed that rolipram significantly would lead to decrease in phosphorylation level of AKT (Ser-473) in TICs (P<0.003) and U87 MG cells (P<0.027) relative to corresponding control. Pretreatment with SC79 (4 μg/mL) significantly counteracted rolipram-induced reduction of phosphorylated AKT (Ser-473) level in TICs (P<0.041) and U87 MG cells (P<0.003). Interestingly, IC50 of rolipram in the presence of SC79 (4 μg/mL) significantly increased the relative level of p-AKT (Ser-473) in U87 MG cells (P<0.026) but not in TICs (P<0.1) as compared to corresponding control (Figure 5 C). IC50 of rolipram significantly reduced the relative level of MMP2 in TICs (P<0.01) and U87 MG cells (P<0.006) as compared to control.

The relative expression value of MMP2 was not significantly altered following incubation of the cells with IC50 of rolipram in the presence of SC79 (4 μg/mL) relative to control (U87 MG cells: P=0.711; TICs: P=0.573). The relative level of MMP2 reduction caused by rolipram was rescued by the pretreatment with SC79 (U87 MG cells: P=0.002; TICs: P=0.032). The relative level of intracellular VEGFA was significantly decreased by rolipram at IC50 in the presence (TICs: P<0.003; U87 MG cells: P<0.001) and absence (TICs: P<0.01; U87 MG cells: P<0.01) of SC79 (4 μg/mL). The relative level of intracellular VEGFA reduction by rolipram at IC50 was significantly more in the absence of SC79 than that in its presence (U87 MG cells: P<0.01; TICs: P<0.05) (Figure 5 D).

## 4. Discussion

In the current study, we could find the cytotoxic action of rolipram on primary culture of TICs and monolayer culture of U87 MG cell line. We found that rolipram imposed its cytotoxic effect through inhibition of AKT phosphorylation. Stimulation of AKT activation by SC79 considerably decreased cytotoxicity rate induced by rolipram in GBM cell cultures. Interestingly, pretreatment with SC79 in rolipram-treated cells had an inhibitory effect on cell growth in comparison with the control. However, this response was not significantly seen in TICs. Our data clarified that in SC79-pretreated TICs, rolipram could not yield a sensible change on BCL-2 expression, in accordance with the non-significant cell viability suppression by rolipram plus SC79. In other words, despite the central role of AKT pathway in rolipram-induced TICs survival suppression, induction of cell death by rolipram was mediated via AKT dependent/independent pathways.

To our point of view, inhibition of PDE4 by rolipram can abolish AKT phosphorylation level and results in cell growth regression through two mechanisms; proliferation inhibition and apoptosis induction of U87 MG cells and TICs. Apparently other molecular pathways might be involved in these procedures. A possible explanation of these results is that cell growth inhibition is also mediated via PKA and exchange protein directly activated by cAMP (Epac), direct effectors of cAMP, which stand on the top of AKT and MAPK signals. PDE4 inhibitor elevates the intracellular level of cAMP, and subsequently either inhibits AKT kinase independent of PKA and Epac (Smith et al., 2005), or switches the PKA stimulatory activity to inhibitory pattern of activity, or activates Epac and consequently results in cell cycle arrest (Chen et al., 2002; Moon et al., 2012; Sengupta et al., 2011).

It is also suggested that cAMP-dependent AKT inhibition is mediated through protein phosphatase 2 (PP2A) triggered by Epac signal (Hong, Lou, Gupta, Ribeiro-Neto, & Altschuler, 2008). Besides, it was found that cell death is due to elevation of cAMP by its analogues results from inhibition of AKT and extracellular signal-regulated kinase (ERK) pathways (Wang et al., 2001). We confirmed that AKT had a central role in cell growth-inhibitory effect of rolipram; however, it is supposed that multiple signals such as PKA, protein kinase C (PKC) (Sengupta et al., 2011), ERK, and AKT might be involved in cell growth repression caused by PDE4 inhibitor.

We found that despite substantial induction of cAMP level in TICs treated with rolipram plus SC79, sensible decrease is not seen in cell survival and proliferation. These controversial results challenge the hypothesis of PKA/Epac-mediated cell survival inhibition following treatment with rolipram in the presence of SC79. Thus, it is conceivable that apoptosis of TICs was mediated via an independent intracellular signaling pathway which is different to the pathway involved in TICs survival suppression. Therefore, more studies are required to elucidate the mechanism of cAMP-mediated apoptosis of TICs by rolipram. Based on the results, the effect of rolipram on the reduction of MMP2 level became inconspicuous according to increase in p-AKT (Ser473) level when SC79 was added to cultures before rolipram.

However, intracellular level of VEGFA was not relevant to the phosphorylation level of AKT in rolipram-treated cultures pretreated with SC79. Interestingly, a significant decrease in endogenous VEGFA level was observed despite increase in AKT phosphorylation induced by SC79 in rolipram-treated cultures. Therefore, AKT signal exclusively mediates the modulation of endogenous MMP2 but not VEGF by rolipram, and AKT signal transduction pathway may play a crucial role in rolipram-induced modulation of VEGFA level but it is not the only mediator in this process. Apparently, other unknown mediators contribute in regulating the intracellular VEGF level by rolipram. Recent data indicate that PDE4 could enhance hypoxia-inducible factor (HIF) signaling in lung cancer cells through PDE4-cAMP-PKA axis and induce VEGF secretion.

Available evidence supports the existence of a crosstalk between PDE4 and HIF. Indeed, data have elucidated that hypoxia and subsequently HIF activation would lead to increase in PDE4 activity and vice versa. Hence, PDE4 inhibitor might reduce intracellular VEGF level by blocking the PDE4-PKA-HIF axis in addition to blocking AKT signaling pathway. We detected high levels of PDE4A and VEGFA in core-derived TICs that supports the theorem of PDE4-mediated VEGF induction. Moreover, the peripheral zone of GBM is either devoid of TICs or rarely represents markers of TICs and often portrays similar characteristics to normal brain cells (Pistollato et al., 2010). Hence, lower quantity of VEGFA mRNA and PDE4A protein in peripheral zone is certainly because of TICs absence in this area. Therefore, targeting PDE4 with rolipram to improve the patients with GBMs overexpressing PDE4 and VEGF, results inspire more future studies.

In conclusion, we introduce that anti-proliferative and pro-apoptotic consequences of rolipram on GBM cancer cells are mediated via AKT dependent/independent mechanisms. However, some heterogeneous responses of different GBM cells to same treatments were seen in this study. Downregulation of MMP2 but not VEGFA levels due to PDE4 inhibition was dependent on the blockage of AKT activation. Hence, the necessity of doing a detailed study on the possible signal transducers involved in cytotoxicity caused by rolipram is felt. It is suggested that an extremely accurate survey is carried out on the invasion, tumor and vascular growth regression by rolipram and its co-treatment with AKT inhibitors in glioma tumor-bearing models. Additionally, our data encourage designing the clinical trials in the future to determine the effective and tolerable dose of rolipram in patients with GBM.
